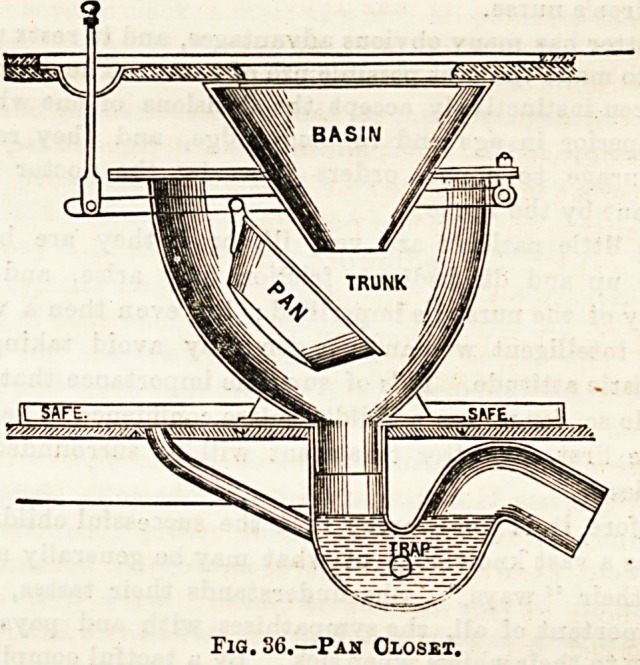# "The Hospital" Nursing Mirror

**Published:** 1896-09-05

**Authors:** 


### "The Hospital" Nursing Mirror.

**Being the Extra Nursing Supplement of "The Hospital" Newspaper.**

[Contributions for this Supplement should be addressed to the Editor, The Hospital, 428, Strand, London, W.C., and should have the word "Nursing" plainly written in left-hand top corner of the envelope.]

### News from the Nursing World.

### ROYAL BERKSHIRE HOSPITAL.

An Art Exhibition is to be held in October in the Victoria Hall, Bracknell, in aid of the funds of the Royal Berkshire Hospital, Reading. Her Royal Highness the Duchess of Teck, who with the Duke of Teck, will then be staying at Warfield Park, with the Hon. Arthur and Lady Clementina Walsh, has consented to open the exhibition.

### THE EIGHT HOURS' DAY.

The Victorian Government is seeking to insist upon the adoption of an eight hours' day for nurses at the Melbourne Hospital, an act of coercion which the authorities of that institution are by no means prepared to accept. The very large increase in the nursing staff which the change would necessitate would mean the expenditure of something over £2,000 per annum, besides the immediate cost of new buildings to accommodate the additional nurses.

### ROCHDALE DISTRICT NURSING ASSOCIATION.

The nurses of this association have just moved into new quarters, No. 110, Yorkshire Street. We hope they have met with a satisfactory response to the appeal issued by the committee for help towards the furnishing of the house. Many people who may not be able to send a donation in money can yet make some really useful gift in this way if they will take the trouble to find out what is really needed. On the other hand, it is no help to busy workers to have impossible rubbish thrown upon their hands as being "good enough to give away."

### QUEEN'S NURSES AT BERWICK.

In the report of the Berwick-on-Tweed Ladies' Nursing Association for June and July the executive committee especially record their thanks to Lady Crossman, President of the Association, Lady Jerningham, the Hon. Mrs. Askew-Robertson, Mrs. Milne-Home, and Mrs. Baldwin, vice-presidents, and other friends, for their contributions towards the expenses of the nurses' visit to London in June, when through their kindness "the nurses enjoyed not only the honour of presentation to Her Majesty the Queen, but also a most pleasant week in London."

### PROBATIONERS' GRIEVANCES.

A dispute has recently arisen in relation to the payment of nurses at the Belfast Union Infirmary. Some probationers entered the service of the Guardians understanding that their salary would be £10 for the first year, £12 for the second, and £15 for the third. On inquiry, however, it appears that there is no intention to pay more than £10 a-year unless a probationer is elected a staff nurse. Obviously this is a most unjust arrangement, giving a minimum salary to a nurse who might have four or more years training, without being called a charge nurse. We imagine this arrangement arises from a misunderstanding of the usual customs which obtain in other institutions in respect to nurses' salaries. Probably the scale of payment was copied from the rules of some other institution, and it will be well for the Guardians to inquire how these rules are carried out in the same place.

### MATER INFIRMORUM HOSPITAL, BELFAST.

The new hospital, to securing funds for the erection of which the Sisters of Mercy have been devoting themselves with unflinching energy during many months past, is rapidly nearing completion. The "Hospital Co-operating Association" has done much to help them, and the good Sisters have received plenty of encouragement from the people of Belfast. The new hospital, like the present building, will be open to patients of all creeds. Contributions towards the furnishing fund are being now earnestly asked for, so that the internal arrangements of the new hospital shall be thoroughly complete and up to date.

### TIMELY HELP.

The nurses of the Hucknall District Nursing Association have lately had their hands more than full in consequence of an epidemic of typhoid fever in the neighbourhood. A deputation from the association waited upon the District Council the other day, and begged for some financial assistance to enable the services of an additional nurse to be obtained. It appeared from the explanation of the clerk that the Council was not legally permitted to give such help—at least, that "the law was not precise on the point." Nevertheless, finally a grant of £10 was made. It is a pity that such eminently practical and useful assistance should not be within the discretion of the district councils. It would be difficult to find a cause more deserving of succour.

### NURSING IN IRISH WORKHOUSES.

A meeting of those interested in the Irish workhouse reform movement is to be held in Dublin on October 1st or 2nd, at which Lord Meath has consented to preside. Any information may be obtained from Miss O'Reilly, care of Charles Eason, jun., 80, Middle Abbey Street, Dublin, hon. secretary *pro tem.*

### CONFERENCE OF WOMEN WORKERS AT MANCHESTER.

Many interesting subjects will be discussed at the forthcoming conference to be held in Manchester under the auspices of the National Union of Women Workers. Especially interesting to nurses, of course, will be the paper on "Registration and Employment for Midwives." Amongst the names of speakers on this matter we note those of Dr. Annie Macall and Miss Rosalind Paget. On one day there will be a conference of women guardians, at which papers will be read by Miss Mackee (Marylebone), and Mrs. Hazzledine (Nottingham).

### BERWICK HOME AND HOSPITAL.

The Berwick Home and Hospital for Delicate Girls was formally opened last month by the Right Rev. Lord Plunket, Archbishop of Dublin. The Home was founded by the late Mrs. Berwick for the special benefit of sick and delicate women between the ages of fourteen and thirty—shirtmakers, sempstresses, &c. By her will, her daughter, Miss Emily Berwick, has been appointed president. Women of the class for which the hospital is intended, overtaken by illness or constitutionally unfitted for a life of hard toil and constant struggling to earn enough to keep body and soul together, sadly need a helping hand, and this institution has a good and useful work before it.

### NURSES FOR ADEN.

For some time past the need for a proper nursing staff has been much felt at the European General Hospital, Aden, where the number of in-patients has lately greatly increased, amongst them there frequently being employés of the great steamship companies whose boats touch at Aden. A scheme has therefore been set on foot to provide the hospital with an efficient staff of nursing sisters, for which it is estimated that a capital sum of about £9,000 will be required Anyone caring to contribute to the fund is requested to write to the Political Resident, Aden (Lieutenant-Colonel W. B. Ferris), marking the envelope "Nursing Fund."

### DISTRICT NURSING FUND, DONAGHADEE.

A successful concert was given at Donaghadee on August 28th in aid of the District Nursing Society. The society has only just issued its first annual report, which, it is pleasant to record, shows that, though young in years, its usefulness is being abundantly recognised by the people of the neighbourhood. Much interest is being taken locally in the efforts of the committee to help the poor in their times of sickness and want. A needlework guild has been started in connection with the society, which it is hoped will prove a helpful supplement in the winter when warm garments are sadly needed.

### NURSING IN THE STATES.

The New York Training School for Nurses, attached to the Bellevue Hospital in New York, in its twenty-third annual report, chronicles a year of successful though comparatively uneventful work. The managers of the school, at the request of the medical board, have lately asked for additional allowances for ten extra nurses in order to provide for the due nursing of the alcoholic ward, and to provide for emergencies, to which the Board of Apportionment has consented. The term of training is for two years, during which time complete courses of lectures are given by the medical staff, and classes held by the matron and her assistants. During the first year the nurses serve in the wards of the Bellevue Hospital; during the second they are expected either to remain in the hospital or go to private cases, as the superintendent may assign. The pay for the first year is 7·00 dols a month; for the second, 12·00 dols.

### WOMEN TO THE RESCUE.

The Women's Club, of Utica, New York, is evidently a public-spirited body. By way of improving the state of the streets of Utica, the club has undertaken to bear the expense of having two of the central blocks in the city swept and the refuse removed in bags after the fashion most approved in the best parts of New York. This is to be done experimentally, and if the result prove satisfactory the city authorities will be asked to extend the system.

### BALFOUR HOSPITAL, KIRKWALL.

The annual ball held in the Town Hall, Kirkwall, in aid of the funds of the Balfour Hospital, proved this year to be a complete success, socially and financially. The hall was prettily decorated, through the kindness of Captain Moore and the officers of H.M.S. "Research," with bunting and evergreens by a party of sailors, and the supper and refreshments were supplied gratuitously by some ladies in Kirkwall, with the help of a few subscriptions. The people of Orkney are certainly to be congratulated on the result of their energetic efforts, an enjoyable entertainment, and, after meeting all expenses, a sum of £25 handed over to the hospital trustees. The three balls which have taken place since 1894 have realised rather over £80.

### HIGH-HEELED SHOES AND BANGLES.

"I want a serviceable nurse for one of my private patients." The doctor glanced down the list of names which was offered to him. "Yes, they all seem to be properly trained, but that isn't everything." "May I ask what you mean by 'serviceable'?" said the head of the institution to whom these remarks were addressed. "Well, a woman who doesn't wear bangles, nor high-heeled shoes, and who thinks more of her patients than of her appearance." "I cannot promise you one of that kind at present," said the lady; "such services are always bespoken, and the supply is never equal to the demand. Perhaps you would try one of those on the list I showed you?" The doctor shook his head decidedly; "No, thank you," he said, "I prefer to trust one of my housemaids. If I cannot get a woman whose appearance is consistent with her profession, I must dispense with the services of a trained nurse, and advise the patient to do so."

### SHORT ITEMS.

Queen's College, Belfast, has during the past year thrown open to women for the first time all its scholarships and college prizes. Seventeen women students were in attendance during the year.—It s only within the last few weeks that ladies in Berlin have been permitted by the law to make use of the outside seats of the trams and omnibuses.—An allowance of thirty rupees a month is now allowed by the Indian Government to sisters in the Army Nursing Service for the keep of a horse.—The Earl of Cadogan opened a Bazaar in Cork on Wednesday last (September 2nd), in aid of the Women and Children's Hospital. —The Leamington District Nurses are now happily settled in their new home, 2, Radford Road.—A very successful garden party was held in the charming old-fashioned grounds of Lytham Cottage Hospital the other day.—Miss Lena Evans, 59, Tufnell Park Road, writing in reference to the supply of day or "visiting" nurses for the middle classes, informs us that she has tried this plan with good success for five years past.—The seventh annual flower show was held at Alcester the other day, in aid of various local hospitals.—A well attended open-air fête was recently held at Bramhope, Torquay, by permission of Mrs. Rawson, in aid of the Torquay Sick Poor Nursing Fund.

### Hygiene: For Nurses.

By John Glaister, M.D., F.F.P.S.G., D.P.H.Camb., Professor of Forensic Medicine and Public Health, St. Mungo's College, Glasgow, &c.

### XXII.—THE SANITARY FITTINGS OF THE HOUSE (*continued*).

Kitchen and Pantry Sinks.—These are usually made either of cast-iron, plain or enamelled, or earthenware. They ought to be sufficiently capacious, of oblong shape, and the grated or perforated waste-pipe opening ought not to have too large holes, lest débris of food descend and block the pipe. They ought to have a "waste" and an "overflow" pipe, and

Fig. 35.—Buchan's Grease Trap.

should not be surrounded by woodwork. From kitchen operations greasy matters find their way by the sink into the waste-pipe, and, when cooled, are liable to block the pipe. To prevent this a special form of trap has been devised. One of the best is Buchan's grease-trap, of which the above (Fig. 35) is a figure: A, is the inlet pipe from sink—the arrows indicate direction of flow; B is the grease-chamber, in which fatty matters solidify, and which may be removed by the movable lid, E; C is the outlet pipe, by which grease-free water escapes into house-drain; D and P are openings by which the trap may be cleaned; The congelation of fat is effected by making the inlet-pipe higher than the outlet, and the tongue of the trap nearest the outlet lower than that nearest the inlet. Its operation is very efficient.

Main Waste Water Pipe.—This pipe, into which all branch pipes from previously mentioned fittings enter, may either pass downwards on the inside of the house wall, or on the outside. Most practical men are of opinion that, provided the pipe makes its exit from the interior to the exterior of the house above the ground level, and that it be disconnected from the house drain, either by opening over a trapped gulley or into a ventilating trap, no objection can be offered to placing it on the inside of the house wall. In this position it is less liable to freeze, and it is less subjected to alternations of heat and cold, which slacken joints and warp pipes because of the alternate expansion and contraction of the metal. To prevent freezing it is customary to keep the water dribbling in this pipe, but this often helps what it is intended to prevent. A thin layer of water passing down a pipe which has a northerly or easterly exposure, will quickly freeze in its course during severe frost. This, by gradual accretion, soon blocks the pipe. It is an open question whether the waste-pipe should open over a grated gulley on the ground level, or into a ventilating trap below the ground level. In either case the trap prevents the return of harmful products from the house drain. The determining factor ought to be the exposure of the pipe in respect of the points of the compass. If it be the south or west, the open method is preferable; if the north or east, the closed, because of the liability of the open gully becoming filled up with ice in frosty weather, and of the water from the waste-pipe being diverted on to the surface of the yard or pavement.

Rain-water Conductors and Courtyard Gullies.—These practically belong to the waste-water system. Sometimes the rain conductor is formed by the simple extension of the waste-pipe to the eaves, where it is joined to the eaves-channels. In many cases, however, they are, necessarily, separate fittings. Whatever their position, they must be trapped at their junction with the house-drain. Courtyard gullies carry off surface water. They are made of special form so that mud in suspension is allowed time to settle, when it may be removed periodically. From what has been said, the following principles may be laid down, viz.: (1) That all fittings intended to convey waste water should be connected with a main waste pipe only; and (2) there ought to be no connection with the waste-water pipe of any fitting for the conveyance of excretions. Hitherto this has not been general. It is by no means uncommon to find both classes of fittings united in the same main pipe. But waste-water fittings should only be united to a main waste-pipe, and soil fittings to a soil-pipe.

Soil-Pipe System.—The fittings which belong to this system are: (1) the water-closet and slop-closet; (2) the urinal; and (3) the main soil-pipe.

Water-Closet Apartment.—Much too little consideration has hitherto been given to the place occupied by this sanitary convenience. It is but far too common to find it placed in a cramped, dark, ill-ventilated apartment. Probably the reasons for this were want of thought and ignorance of the relation of drain connections and certain filth diseases. Hygiene has demanded more suitable conditions, and they are gradually now being realised. For tenement houses of the poorer class, one closet serves for three or four families, and in flatted houses of a better type there is a closet for each family. In either case, ventilation is but ill accomplished. In such circumstances the apart-

Fig. 36.—Pan Closet.

ment is poorly lighted by a small window opening out upon a common stair-landing. When this window is opened the current of air instead of being from the closet outwards, as is expected, is quite the reverse, owing to the difference of temperature of the interior and exterior of the house. This, obviously, is very objectionable. Indeed, it may be fearlessly said, that while there is no apartment of a house which more greatly demands free ventilation than this, there is none which receives less. The system most suitable for tenement closets is that denominated "cross" ventilation, which is secured by providing windows at opposite sides of the apartment and at right angles to the doorway. Compatible with comfort, every closet should always be wel flushed with a continuous current of air.

The closet itself now requires to be considered. During the last twenty-five years this fitting has undergone an evolutionary process—one might well call it a revolutionary process. The process, however, has been slow, for a variety of reasons. The first closet—a valve closet—was patented in 1775 by Alexander Cummings. Bramah, the famous mechanician, also invented a valve closet. Since then many patents have been taken out by many inventors. The closets in use to-day may be divided into four typical classes, viz.: (1) the valve-closet; (2) the pan-closet; (3) the hopper-closet; and (4) the "flush"-closet.

The valve-closet has now, except in the case of one or two specially good forms, fallen into disrepute. Its chief merit is that the contents are suddenly projected, on release of the valve, into the trap; and its principal demerit is that the seat of the valve—which must be accurately fitting—is liable to be dislocated by any substance which gets fixed therein, thereby allowing the water in the basin to gradually drain away. They are thus very liable to get out of order.

The pan-closet is still largely in use, but is giving way gradually to a less complicated and much safer form of closet. It is composed of the following parts, viz.: (1) An earthenware basin; (2) a copper pan which closes the bottom of the basin in which the water is retained; (3) the receiver, or container, or trunk, composed of cast iron, into which the basin and copper pan are are fitted; and (4) the trap. Fig. 36 illustrates this form of closet, as used in Scotland. The handle, seen on left of figure, operates at the same time to open the cistern for flush-water, and to depress the pan, thus emptying and flushing the basin at one pull. Of this closet an American writer once said, "It probably was not, although it might have been, the invention of the Devil." It is, certainly, a most unhygienic fitting, first, because it is composed of parts that cannot be cleaned; and second, and chiefly, because the sides of the "trunk" become coated with decomposing excreta, the odour of which is projected into the apartment each time the handle is pulled. English writers have urged an additional objection, viz., that the trap used is a D-trap. In Scotland, however, this objection has no force, since the Siphon, or S-trap, is alone used.

### Trained Nurses' Clinic.

### XII.—THE NURSING OF CHILDREN—(*continued*).

The responsibility attendant on the nursing of children cannot be over-estimated; neither can it be escaped. It may be possible to shift on to the shoulders of an adult patient the blame of an injudicious action; but this cannot be done in the case of a child. If the grown-up person be of weak intellect, or be rendered by the nature of his illness incapable of exercising due discretion, he must, of course, be guarded from foolish acts by the nurse. In such a case her responsibility is as great, and her position more difficult, than that of the children's nurse.

The latter has many obvious advantages, and it rests with herself to make the best possible use of them. The majority of children instinctively accept the decisions of one who is their superior in age and in knowledge, and they rarely have courage to oppose orders given by the doctor and carried out by the nurse.

If the little patients are very ill, or if they are badly brought up and disobedient, friction may arise, and the authority of the nurse be imperilled; but even then a well-trained, intelligent woman can generally avoid taking an antagonistic attitude. It is of supreme importance that she should do so, for unless a child's entire confidence is gained from the first, his after treatment will be surrounded by difficulties.

Therefore it is noteworthy that the successful children's nurse has a vast knowledge of what may be generally specified as their "ways." She understands their tastes, and, most important of all, she sympathises with and pays due attention to their whims when sick. By a tactful compliance with these fancies, she secures the grateful affection of the small invalids, and eventually finds that obedience is willingly rendered on the rare occasions when it becomes necessary to enforce it.

One of the secrets of a wise nurse's influence can be traced to the fact that she gives very few orders, and always insists on implicit obedience to those she does give.

The constant irritation caused to children by an opposite method is most harmful to their tempers. Incessant worry is quite as trying to juveniles as to adults, and such a condition is inseparable from the repeated "Do this," or "Don't do that," with which many well-meaning persons harass little people.

"She never worries," is a phrase which implies, in a patient's mouth, very high approval indeed, because it conveys the impression that the skill of the nurses is not discounted by any undue fussiness. "Do not worry," is a motto specially applicable to a child's nurse, or at least "Worry yourself, if you must, but never worry a child."

Many worthy folks fancy they understand children, because they make those they know well happy in their presence, but the wide comprehension of the many-sided natures of the little ones necessitates the possession of a living and loving sympathy with all phases of childhood. The unattractive, morose, and, alas! often misunderstood child has a deeper claim on the good offices of the sick nurse that the sunny tempered and loveable darling who retains, even in illness, her attractive personality.

The good children's nurse has a place in her heart, and a thought in her head for every one who comes within her scope. Each sick child has a special claim, which she cheerfully acknowledges. Her whole life seems absorbed in the war which she wages against disease. The casual observer says, "Poor little sufferer," and passes on, feeling helpless in the presence of the sick child; but the capable nurse looks with distaste at such an abnormality. She takes the child to her heart, but against the disease or injury from which it suffers she wages immediate and incessant war. She sets herself to second every effort of the doctor, and will never rest content till the young patient has been transformed into a normally healthy child. If the case seems hopeless, the little life will be fought for unweariedly; and should death be remorseless, and claim its young victim, then, and then only, will nurse own herself beaten.

The wonderful recoveries of sick children justify the nurse's confidence. She watches the light of life and the glow of health return to the frail creature who has been considered an almost hopeless case for days, and she triumphs at each sign of improvement. With what pride does she point them out to the doctor, and how those two rejoice at each little life which is rescued by them!

(*To be continued.*)

### Appointments.

Wilhelmina Hospital, Amsterdam.—Miss L. Kruysse has been appointed lady superintendent at this hospital, vice Miss Raeynvaan, who has been compelled to resign her position owing to ill-health. Miss Kruysse's name is well known as that of a pioneer in district nursing in Holland. She studied nursing for three years at the Royal Infirmary, Edinburgh, and then specially trained for district nursing at St. Patrick's Home, Dublin, the Irish Branch of the Q.V.J.I. She began her work at Zwolle in 1894, and has since extended it to Amsterdam and Rotterdam. We cordially wish Miss Kruysse all success in her new and important position.

### Nurses in 1896—Their Quarters, Hours, and Food.

### THE LONDON HOSPITAL.

### I.—Terms of Training.

Before being admitted to the wards on a month's trial would-be London Hospital probationers are now required first to enter the Preliminary Training Home in connection with the Hospital for a six weeks' course of instruction in the ordinary routine of ward work and in elementary physiology, anatomy, hygiene, and sick-room cooking. The London Hospital Training School has been the first in the metropolis to try an experiment of this kind. It has now been in operation for rather more than a year. Examinations conclude the course, after which suitable candidates are transferred to the wards, the three years' engagement ultimately signed counting from the day they enter the hospital. Certificates are given at the completion of the full term of two years' training, but a third year's service is required from those who are accepted as regular probationers, and the remuneration for that year is in accordance with the position the nurse is selected to fill. Paying probationers are admitted for periods of three months on payment in advance of thirteen guineas. In work and training no difference is made between non-paying and paying probationers. Day and night duty is taken in alternate periods of three months. Examinations are held yearly, and prizes are given. Regular probationers are admitted between 25 and 35 years of age. Paying probationers must be not under 22 nor over 40 years.

### II.—Hours of Work and Times Off Duty.

Nurses are on duty from 7 a.m. to 9 p.m., with two hours daily off duty, occasionally four, and one half-hour after the morning's work. Half an hour is allowed for dinner, and "sufficient time for tea at a convenient hour." Night nurses and probationers are on duty from 9.20 p.m. to 9.20 a.m. Sisters come on duty in their wards at 8 a.m. to 10 p.m., with two hours off during that time. From the printed timetable it would seem that the hours of sleep are somewhat curtailed for the nurses generally—especially in the case of night nurses. These are called at 8.15 in winter and 6 p.m. in summer; they are on duty, as stated, from 9.20 p.m. to 9.20 a.m., being off duty from 10.30 a.m. to 1 p.m. in winter, and from 6 30 p.m. to 8.30 p.m. in summer, having to be be in bed by 1.30 p.m. and 11 a.m. respectively. They are, therefore, only in bed for six and three-quarter hours in winter and seven in summer. The day nurses are rather better off, being in bed from 10.30 to 6 a.m., but every nurse should be able to have at least eight hours' sleep every night. Two hours daily off duty is the rule at the London, the time at which it is taken, within limits, being at the discretion of the sister of the ward. It is also in her power to allow a four hours' pass occasionally. Probationers are given a day's holiday from 10 a.m. to 10 p.m. every month, and a week's holiday at the end of each six months, being allowed a full month's holiday at the end of their two years' training before entering upon their third year's service on the permanent staff. Staff nurses are off duty every fourth Sunday from 9 a.m. to 10 p.m., and a half-day is given every month from 1.30 p.m. to 10 p.m. Late passes for concerts or theatres are allowed on days off. Sisters are off duty every day from 5 to 7 p.m., and have one whole day and one half day each month. Staff nurses have three week's holiday in the year. Sisters one month. The advantage of a "day off" is very much diminished where, as at the London, nurses have to come on duty in the morning. Undoubtedly the nurse ought not only not to come on duty at all, even for an hour, but she also should not be expected to appear at the half-past six breakfast, in order that extra time in bed may fit her for getting the best possible result from her hard-earned holiday.

### III.—Meals.

The Nursing Home is presided over by a home sister, who controls the housekeeping arrangements under the matron, and is responsible for the servants. The food provided for the nurses is excellent in quality, and much trouble is taken to vary the dietary as much as possible. The day nurses for their half-past six breakfast have tea and coffee, eggs, sardines, potted meats, cold bacon and ham, fish, and so on in turn, and always jam and marmalade. A second breakfast at half-past eight, after the heavy work of the morning is over, is taken in the wards, consisting of tea and bread and butter. The dinners of day and night staff are of hot joints, two vegetables always, and puddings of various kinds, with fish on Fridays for those who wish it. Milk, soda water, ale and stout are provided at every meal, and in summer lemonade. Tea the nurses make for themselves either in their wards or in their own rooms, allowances of tea, sugar, butter and bread being served out, the two first once a week, butter twice, and bread every day, a bad custom, leading to food being kept in cupboards in the wards, exposed to all the contamination of hospital atmosphere, and prepared in a scrambling and altogether unfitting way. Supper consists of soup, potato pies, sausages and potatos, fish or puddings. The night nurses' breakfast at 8.50 p.m. is a meal which varies a good deal with the season of the year, cold meat and salads being preferred in summer and hot pies or fish in cold weather. Night nurses take provisions for the night with them from the dining-room—eggs, bacon, cold meat, or meat pies, with bread and butter—taking their night meals as they find convenient in the ward. The ward sisters have breakfast, lunch, and tea in their own rooms, the ward maid fetching supplies from the home. They dine all together in the sisters' dining-room at seven o'clock, a meal at which Miss Lückes sometimes presides.

### IV.—Salaries and Uniform.

Regular probationers are paid for their first year at the rate of £12 per annum; for the second, £20. After appointment on the permanent staff, nurses on day duty are paid at the rate of £22 the first year, rising £1 annually to £25. Night nurses pay is at the rate of £24, rising annually £1 to £27. Sisters salaries vary from £40 to £60 a year, according to the number of beds under their charge. Material for four print dresses for the Sisters, three for the nurses and probationers, and three caps are provided during the year. Aprons are not given by the hospital. Outdoor uniform is not compulsory, except for members of the private staff for whom it is provided. The adoption for the Sisters of washing dresses of a particularly pretty shade of blue linen is a much-to-be-commended improvement upon those of dark blue cashmere formerly worn. These dresses are sent to the hospital laundry, and an allowance of 2s. 6d. a-week is granted to all the staff for washing.

### V.—Nurses' Quarters.

The London Hospital possesses the best nursing home of any of the metropolitan voluntary hospitals. It is an entirely separate building adjoining the hospital, with access to the wards on each floor, or by way of the garden. Separate and good-sized bed-rooms are provided for the nursing staff; there are dining-rooms for the sisters and nurses respectively; a pleasant sitting-room for the nurses and probationers which contains an ample library, and also an excellent collection of specimens, prepared and presented by members of the surgical staff of the hospital. On the top floor of the home is the sick-room, with several beds, presided over by a permanent nurse in charge; here nurses are sent for slight ailments. Cases of serious illness are treated in the wards, two small rooms with one bed each being especially kept for their reception, leading from two of the wards. On each of the five floors of the home are bath-rooms, lavatories, and a small scullery provided with gas kettles, which are kept boiling during the afternoon for nurses off duty to make their tea when they like. The night staff are lodged in cubicles on the top floor of the hospital itself. Up to the present the nurses belonging to the private nursing institution have found accommodation in three houses just outside the hospital gates. An additional wing is now being built to the home, which will provide ample accommodation for the whole of the staff. The ward sisters at the London Hospital have bed and sitting rooms off their wards, in some cases only divided from the ward by a mere partition, a bad arrangement, which obtains in many of the older hospitals, and is calculated to interfere with the comfort, health, and sleep of the sisters. The Home Sister and Matron's assistants have pleasant bed-sitting-rooms in the home. Tredegar House, the new preliminary training home in Bow Road, accommodates twenty candidates at a time. The house has lent itself well to adaptation; the rooms are charmingly furnished, as, indeed, are all the nursing quarters at the London, and there is good provision in the way of study and class rooms, with a pleasant dining-room.

### A Holiday on Robben Island.

From a Correspondent.

It is the impression of a good many people, I believe, that Robben Island is the gloomiest, dreariest, most melancholy spot on the face of the globe, and that it is only inhabited by lepers, who pervade the whole island, and whose woeful forms may be encountered at every turn.

To those who thus picture the island in their own minds I can only say, Go and see for yourselves; or, if that is impossible, listen to my tale of a holiday I once spent on Robben Island. I remained there a fortnight, I enjoyed myself all the time, and I never saw the lepers except when I went to the leper hospital for the express purpose.

To begin from the beginning, if the weather is propitious and the sea calm, the half-hour's trip in a little steamboat from Cape Town Docks is in no way objectionable. An order has to be obtained from the Colonial Secretary before a visit can be paid to the island, as the whole place is, so to speak, one big Government institution. At the time of my visit here was not, as there is now, a pier, and owing to the extreme shallowness of the water the landing had to be effected in the following manner: First, you descended from the steamboat into a rowing boat, which brought you somewhat nearer to land, and then you were met by a party of convicts wading up to their knees, who conveyed the ladies in sedan chairs, and the gentlemen a "pick-a-back," safely to shore. Since the erection of the pier I conclude that we have changed all that.

Robben Island is made of sand, but then so is the whole of the south coast of Africa so far as I have seen. The name is derived from a Dutch word meaning seal, as the island was in the early days of the colony a seal fishery. There are none there now, but I saw not long ago a small seal that was washed ashore near Port Elizabeth.

There is not such a thing as a bush or a tree on the island, yet the surface is green with a sort of low shrub, and at one season of the year it is white with arum lilies. There are over a thousand inhabitants, including the lepers, the lunatics, and all the Government servants employed in the various institutions. On one side of the island, facing towards Cape Town, is the village, including the church, the school, the store, the various residences of doctors, chaplains, and so forth. The male leper wards are quite away from the village, and further away still are the female leper wards, which are shut in with a high wall, the female lepers being segregated. Unless, as I said before, one has an interest in visiting the lepers there is no need to see or hear anything of them.

I was myself staying with the matron of the lunatic asylum, whose quarters are as private and cosy as the most fastidious matron could desire, and provided, moreover, with a lovely verandah, on which one can happily sit for a whole day, looking over Table Bay. The asylum is not remarkable for anything except a beautiful wide corridor, than which I have not seen a better anywhere. The patients are confined only by a wire fence, for, as they cannot run away anywhere, except into the sea, it is possible to allow them much more liberty than on the mainland. It always seemed to me as if the view—the wide open stretch of dancing waves, right away to the horizon, on three sides, and on the fourth side the high majestic form of Table Mountain, ever standing in changeless repose—must have a soothing effect on a tired brain, and even a healing effect on a distracted one.

At that time there were new leper wards in the course of construction, and a temporary line was laid from the village to the wards, along which trucks loaded with bricks and other materials ran backwards and forwards. One of my chief amusements was to ride in an empty truck as far as the nurses' home, where any of the nurses who were off duty would join me, and we would go on to the end of the truck line, and then scramble over the rocks on the shore, feasting our eyes on the ocean landscape (!), which never palled upon us, and startling thousands of sea-birds with which the rocks literally bristled.

The island is seven or eight miles (I forget which) in circumference, and for a good walker it is a very pleasant walk round the shore. Heavy rain, which fell on the last two or three days of my visit, prevented me from accomplishing this, and consequently I did not see an old wreck which is on the further side of the island. I paid a very interesting visit, however, to the lighthouse, which is a large one and well worth seeing.

Of course there is a sad side of life on the island. Where is there not? To my mind no lot, not even a lunatic's, is so sad as that of the leper. The lunatic is very often unconscious of his own calamity; but the leper, cut off from wife and children, home and friends, victim of a loathsome disease, yet in full possession of his intellectual faculties, his natural desires and affections, descends into a living grave, in which he may have to spend years. And when all has been done for them that can be done, what does it amount to? Wherever we go in this world, however, we shall "find trouble and heaviness," and we shall gain nothing by shutting our eyes to it.

In fine, a visit to Robben Island is sure to be full of interest to any intelligent person, because the place is, in many respects, unique; and if the intelligent person also meets—as he surely would—with the same kindness, friendliness, and hospitality that I did, his visit will be also full of enjoyment.

### Minor Appointments.

Felsted School Infirmary.—Nurse Maria Elvin has been appointed Head Nurse. She was trained at Great Yarmouth Hospital, having since worked as district nurse at Birmingham, and held the post of head nurse at the Borough Hospital, Bolton, and at the Isolation Hospital, Blaby.

Dorset County Hospital.—Miss Jessica D. A. Earle has been appointed Sister at this hospital. She was trained at the London Hospital, where she afterwards held the post of staff nurse. Since leaving the London Miss Earle has acted as night sister at the County Hospital, Ayr, N.B. She has our best wishes for success in her future work.

### A Book and its Story.

"ONE OF GOD'S DILEMMAS."*

The least good part of Mr. Allan Upward's book is the title which he has chosen for it. Of course, as its name suggests, the story is of a metaphysical order—problematical—though to minds other than the author's this problem is less of a divine than of a human nature, and the "dilemma" in which the several characters in the last of the Pioneer Series find themselves is one which two of them might fairly have foreseen, and for which they, themselves, are clearly responsible. Mr. Allan Upward possesses in a marked degree the art of compelling his reader's interest. His story opens at an out-of-the-way seaside resort somewhere on the English coast, on which the name of Shorwell is bestowed, and here, a stranger wandering along, comes on a group of lads at cricket. With one of the players, impelled by some unaccountable impulse, the stranger entered into conversation. The boy took stock of the man who thus addressed him. "His experienced regard at once revealed to him that his companion was of the tribe or denomination of visitors. Only visitors went about in Norfolk coats and knickerbockers, and carried newspapers down on the front to read." The boy turned away his eyes with a slight touch of disdain. "Etienne had lived in Shorwell all his life, and the inhabitants of Shorwell naturally looked down on visitors. Their presence in the little town was not exactly unwelcome; from a business point of view it was well to encourage them, but, nevertheless, they suffered from the brand of social and intellectual inferiority. Still, Etienne could not forbear acknowledging to himself that this man was not one of the common run of visitors. His face was too dark not to have been exposed to a foreign sun. His whole air, in short, was that of one who had seen life, and perhaps had had an adventurous career."

The new arrival was evidently bent on conversation. "You don't happen to be living here, do you?" he questioned.

"Yes. I have lived here as long as I can remember."

They conversed for some time. The boy talked of his life, his love of cricket and boating. "Perhaps your mother will let you come out with me some time," the man suggested. "Let me see, what is your name?"

"Etienne Bere." "You can tell your mother that my name is Strange, and that I am staying at the Esplanade Hotel." On parting the two new friends arranged to renew their acquaintance—during the following days—on the seashore.

Then the boy went back to his mother, and told her of his adventure.

"He is awfully rich," Etienne explained. "He is staying at the Esplanade Hotel; he is going to hire a yacht and go out every day, and he asked me to come with him. Wasn't it good of him, mother?"

The mother's thoughts that day were engrossed with graver things, the purport of which, later on, she divulged to the boy.

Up to this point in the young boy's career he had lived in ignorance of his antecedents; his mother was a lady, whose reduced circumstances necessitated her keeping seaside lodgings, and his father was dead; much more than that, the child did not know, and now, it was about this father, whom he had never known, that the widowed mother said she wished to speak.

It struck him for the first time as a somewhat strange thing, that his mother had been silent all the fourteen years of his life about the man to whom he owed his existence; and in the elated state of his mind, the boy started when the mother explained that what she had to say would "shock and grieve him."

* "One of God's Dilemmas." Allan Upward. Pioneer Series. (London: William Heinemann. 1896.)

Etienne had been brought up to believe himself a gentleman, he held his head high on this account, and now he was suddenly informed that his father was not a gentleman, that is to say, his people were "not gentlefolks"; he lowered his head in silent resentment at this knowledge—the woman's task became harder as she went on. "His father was the son of a man who owned some fishing-smacks," she continued, "and he had sometimes been out in the boats himself, but generally he spent his time loafing about the town, or in the summer amusing himself among the visitors, and that was how I came to make his acquaintance in the Channel Islands, where they both lived."

Then the widow went on to explain how she had recently become a "Christian, and was helping in some open-air services on the beach, when Etienne's father came to them, pretended to be impressed, and led me on to think I had made a convert of him from Romanism."

The end of it was, as she told the child, that the two were married without the knowledge of anyone but their own two selves. Their married life was one of the shortest duration, details of which Agnes Bere gave to her hearer with the quiet remorselessness of a good woman. Husband and wife had separated, she explained to her son, after a few short days of mutual wretchedness and extremest poverty.

The man went off to make a fortune. The woman, taking advantage of her momentary liberty, came to a quiet place of hiding at Shorwell, wishing never to see her husband again; then the child of the hasty marriage was born. Such was the substance of the widow's tale, told to the listening boy One more revelation—that she was no widow, but that Etienne's father was still living. And it was the news of his being alive which had but just reached her ears. Hence her distress.

The woman's hatred of her husband forms the theme of much of the book—a hatred which only grew when she sees the effect produced upon her son by the knowledge of his parentage.

The boy drowns his care, so to speak, in the excitement offered him in the new friend's presence—the rich stranger who lived at the Esplanade Hotel. Their meetings are of daily occurrence, and the glamour of the elder man's singling out of Etienne ensnares the boy, who looks upon this as chance. After some chapters devoted to a charming description of the friendship existing between the two, and then the *dénouement*, one hardly unexpected, appears at length in the discovery that the rich stranger is the beautiful widow's husband, returned improved in circumstances and strengthened in character to lay his fortune at his quandam wife's feet.

Why the woman did not encourage the advantageous offers made to her by her now exemplary husband, we are at a loss to understand, though it is part of the disagreeable qualities of her character that she cannot estimate the reformation which time and sustained intention have worked in the man, nor understand how limited is her sense of Christian forgiveness. Her anguish at the boy's affection for the man is clearly and masterfully portrayed, and the final scene in the book, the one dramatic incident, carries out to the bitter end the strange consistency of her attitude towards her husband. Over the body of their drowned boy, man and wife meet, and recognise each other. "What right have you to look at him, or interfere?" the mother cries. "You! You came and tried to rob me of him. My boy, my boy, is dead! Move back, your shadow is enough to blight his dead body. You cannot rob me of that. You shall not meddle with him any more; you have done all you could, and what is left of him is mine!"

Sebastian made no answer. He rose up and left Agnes there, passing out of her life for ever.

### District Nurses and Infectious Diseases.

A DISTRICT nurse asks our advice. She has been instructed by her committee not to attend cases of typhoid on the ground that it is a "highly infectious fever," and while attending to a lying-in case in which puerperal fever has occurred she has been told by the doctor that she must leave the case at once, although she assured him that her colleague would take all other midwifery cases, and that she would take all precautions. The nurse asks, "Do you think my committee have any right to fetter me like this?"

Now, as a question of "right," it is quite clear that a committee have a right to engage a nurse under any such conditions as seem to them good, and that the nurse must fulfil the conditions of her engagement. If, however, we put on one side the question of "right," and consider the propriety of a district nurse undertaking cases of typhoid and puerperal fever, there is much to be said against their doing so while attending to other cases.

We would be the last to suggest that a nurse who observed all proper precautions would be *likely* to carry typhoid fever infection from house to house; nevertheless, we are bound to remember that the precautions necessary are somewhat minute and tedious, and not always easy to observe in the cottages of the poor, while the district nurse's duties are somewhat different from those of the ordinary nurse.

It is perfectly well known that nurses attending cases of typhoid fever do sometimes catch the disease: and the way they catch it is, almost certainly, by the infection of their food by imperfectly cleansed hands. It is easy to say that this is carelessness, but it does happen. Now one must remember that a not unimportant part of the work of a district nurse is the practical teaching of the cottagers in the art of preparing invalids' food, and, in fact, in many cases the actual preparation of the food; and so long as the fact remains that nurses not uncommonly poison themselves, even amid good surroundings, by not absolutely disinfecting their fingers, how can we assure ourselves that a nurse, with only the rudimentary appliances of a cottage at her disposal, shall make certain of not carrying the disease to others? There is, moreover, the patient's side of the question to be considered. Typhoid fever is far too serious a disease to be left to the occasional attentions of a district nurse, and if such cases are not removed to hospital they should at least receive more continuous attention than can be given by an occasional visit.

This applies also to the nursing of puerperal fever, which is a malady far too formidable to be undertaken "on one's rounds" by a district nurse. But in this case, much more than in that of typhoid, the question of infection has to be considered. In this respect the nurse occupies a very different position from that of the doctor. Her relations with the patient are so much more intimate and prolonged than his, and the infection of puerperal fever is so difficult to shake off that we do not think it would be right for a nurse attending such a disease to live with another who was attending ordinary cases of confinement.

The fact that such a question as this has arisen at all only emphasises the wisdom of the regulation of the Local Government Board, according to which a district nurse employed by the guardians to attend ordinary cases is not allowed to attend confinements.

On the other hand, we cannot but feel that a peremptory order for a nurse to leave a case of puerperal fever to the haphazard attentions of friends and neighbours was an unchristian act, and that it would have been far better that means should have been taken to isolate the nurse while she was attending to this case alone than to cast the patient adrift for the sake of the nurse attending to her more ordinary duties.

The fact is that puerperal fever ought to be so rare a disease that on its occurrence, as on the breaking out of a fire, everything else should be made subordinate, and every ordinary rule should be held in abeyance; and there can be no doubt that if a committee accepts the responsibility of employing the same nurses to attend ordinary and puerperal cases, they ought to be prepared on the outbreak of puerperal fever to provide a substitute for the nurse attending it in regard to all other cases, and to confine her to that case alone, isolating her meanwhile from the other nurses. Science says that the infected nurse should be separated from the rest, but humanity certainly demands that the patient should not be neglected.

### The Matron.

I, ONCE on nursing quite intent,
Hospital-wards my footsteps bent,
And called to Matron's office, went—
Saw Miss ——.

Who straightway asked the would-be nurse
If to hard work she was averse?
There's that to do, and much that's worse—
Said Matron.

And can you oft reproof endure?
Keep silence when you feel most sure
Not you, but other's the wrong do'er—
Said Matron.

A nurse's duty's to observe
When best she helpfully can serve;
Courage she needs, and also nerve—
Said Matron.

There's much unpleasant work to do,
To banish dirt devolves on you,
Lessons to learn, all mostly new—
Said Matron.

Then to be truthful and sincere,
Own every error without fear,
Honour and duty hold most dear—
Said Matron.

A punctual habit is desired,
Must be by nurses all acquired,
Though hard when feeling very tired—
Said Matron.

To be obedient, patient, kind,
I trust you have made up your mind,
Nursing's not easy you will find!
Said Matron.

Thoughtful, aye, for other's weal,
Tender with their woes to deal,
With kind words to cheer and heal—
Said Matron.

God's smile will on such labour rest,
Not pining, strive to do your best;
New duties will new powers invest—
Said Matron.

That week with mind on duties set
I entered, obstacles I met;
But I my training don't regret—
Under Matron.

In her we found a friend most true,
Amid perplexing work she knew
For all, the right course to pursue—
Dear Matron.

### Medical Missions in India.

### BANGALORE ZENANA MISSION HOSPITAL.

By Sister Clare.

A short account of this recently-opened missionary hospital may be of some interest. The hospital is in connection with the Zenana Missionary Society, but the money for the building has all been collected privately. The hospital is intended for Mahommedan and Hindu women and children. Mahommedan women are kept in strict "purdah" in this part of India, and they will not go to any hospital unless, when there, they can be quite secluded; and, although we have careful arrangements for complete privacy, only a few as yet have ventured to come as in-patients, and the majority of those we take in are Hindus and native Christians.

The hospital is built in three blobks, which are connected by covered corridors. The building is of stone, and is two storeys in height. In the centre block, upstairs, the missionary workers live, Miss Lillingston, L.R.C.P. and S., and the nursing superintendent. Downstairs is the out-patient department, consulting-room, waiting-rooms, compounding-room, &c. Out-patients attend from half-past seven to half-past nine every morning.

In the east and west wings are the wards. The four larger wards each hold five beds. There are besides three smaller wards and the operation-room. Altogether we can accommodate about twenty-seven patients. As is usual in India, the kitchens are outside in the compound. A row of small whitewashed buildings down one side of our high enclosing wall comprise kitchens, storeroom, nurses' quarters, laundry, and mortuary.

The work in many ways is easier and in some ways more difficult than in England. We have no need of fires in the wards for one thing, which saves much trouble. And the food arrangements are fairly simple. Patients on full diet have their rice and curry every day, twice, with no desire for variety, and low-diet patients have milk and "congées." They will not touch beef-tea or beef in any form. Occasionally a high-caste patient objects to eating any food cooked by us; then we are obliged to allow the relations to bring her food from home twice daily.

The caste system is a great hindrance in many ways. English nurses coming out here find they have to give up hope of ever having wards similar to hospital wards at home. The native people cannot understand our desire for perfect order and discipline, and their ideas of cleanliness are so different to ours that it is a difficult task to train native probationers. They are naturally ignorant of many things that ordinary untrained women at home know, and to teach them to be conscientious, quick, and skilful requires much patience. But we feel it is a very important work, for it opens a new sphere for the native women, who usually look upon marriage as their only vocation.

Indeed, we often wish we could describe to Christian English nurses the large sphere of work there is for them in India. Besides the interest of training these women in ways of usefulness, there are unlimited opportunities for teaching the heathen patients the truths of Christianity. A few weeks ago the lady doctor of a mission hospital in a large heathen town asked the writer if she knew of a trained nurse who would be willing to come out and take charge of the nursing, and to train nurses. It was sad to be obliged to say "no."

The different languages are rather a difficulty with us. Sometimes daily prayers have to be taken in three different languages. We have Hindustani-speaking patients, Tamil, Telegu, and Canarese.

One gets very fond of some of the patients. Thiamma was one, a bright, pretty girl of good caste, whom we came to love very much. She was only eighteen, but had had two children. Her eldest, a fat, brown morsel of a boy, aged four, used to come every day to see her, brought by the grandmother. It was a very sad case of multiple caries. Amputation of one foot was advised by the doctor, but the relations would not hear of it. They have a great horror of "cutting operations." Thiamma's old father came several times to make sure it would not be done without his permission.

The brown babies are very fascinating, but now and then we have too many in at once, and the noise becomes somewhat trying! Sometimes we have to allow a woman to bring her youngest child in with her, and this makes an extra baby to wash daily, which the nurses do not much appreciate. Happily not much dressing is required in this country! Since the opening of the hospital, not quite four months ago, we have had sixty in-patients. Of these a good many were maternity cases. We have a confinement, on an average, once a week. Ten of the sixty were gynæcological cases; others have been malaria, burns, broncho-pneumonia, &c. Sad to say we have had five deaths, four of them within thirty-six hours of admission. It is a great pity that so often in this country patients wait until too late before coming to be treated.

### Everybody's Opinion.

[Correspondence on all subjects is invited, but we cannot in any way be responsible for the opinions expressed by our correspondents. No communications can be entertained if the name and address of the correspondent is not given, or unless one side of the paper only be written on.]

### PROMOTION IN PROVINCIAL HOSPITALS.

"Justice" writes: In last week's *Nursing Supplement* a "Provincial Nurse" complained of the system of promotion at one "of the largest provincial hospitals," apparently with some reason, for it is certainly hard lines for the nurses trained in its wards that in six years two only should have been promoted to the post of sister, while there were eighteen vacancies during that time. It is surely a mistake, however, to look at the matter from the point of view only of the disappointed nurses. The infusion of new blood, so to speak, is very necessary now and again, for the world of a country hospital is a limited one, and things are apt to fall into grooves if no outside influence or fresh ideas and ways are introduced. It is undoubtedly for the good of the institution that vacancies on the permanent staff shall sometimes be filled by nurses from other hospitals, though if harmony and contentment are to reign this must be done judiciously and in proper proportion.

### HOUSE DRAINAGE.

"A Correspondent" writes: In the "Mirror" for August 29th there is an article on Hygiene for Nurses containing a diagram intended to illustrate the proper arrangement of the sanitary fittings for a house. I would point out, however, that this diagram is in certain particulars quite out of touch with modern sanitary practice. The main defect is the placing of a trap at the bottom of the soil pipe. Not only is this in itself a perfectly useless complication but it involves putting in a separate ventilating pipe the whole height of the house to ventilate the drain. When an efficient ventilating trap is placed between the sewer and the house drain the latter should have a free run from the trap to the open end above the roof. Then each water-closet cistern is provided not only with a warning pipe but with an overflow connected directly with the drain, an arrangement which no water company would be likely to allow at the present day, although years ago many cisterns were fitted in that way. It is to be noticed, also, that although the so-called anti-syphonage pipes of the w.c.'s are properly taken into an air shaft, which, however, would have been better carried up to the top, those from the sinks and wash-basins are carried into the waste water pipe so close to the waste water delivery pipe as to be practically inoperative. The system most commonly adopted in London flats in recent years is to make the waste water fall pipes in separate lengths, each the height of a storey, and discharge ing, along with the bath and sink pipes of that storey, into the open head of the length below, the topmost pipe being carried above the roof, and it is found in practice that, when hot baths are emptied such steam as is not condensed does not tend to escape at the joints, but rises to the top. As a detail, which is perhaps owing to the diagrammatic character of the woodcut, it should be mentioned that the traps attached to the sink wastes are not represented in the position they ought to occupy. They ought to be as close as possible to the bottom of the sink or basin, so as to leave as short a length of pipe as possible on the room side of the trap. It is curious that the artist should have placed the fresh-water main and supply pipes at a lower level than the sewer and the drain, a condition of affairs which one would hope is very rarely the case.

### The Book World for Women and Nurses.

[We invite Correspondence, Criticism, Enquiries, and Notes on Books likely to interest Women and Nurses. Address, Editor, The Hospital (Nurses' Book World), 428, Strand, W.C.]

Elementary Anatomy and Surgery for Nurses. By W. McAdam Eccles, M.S.Lond., F.R.C.S.Eng., Assistant Surgeon to the West London Hospital. (London: Scientific Press. 1896.) Price .

Probationers will find this a very useful book, giving, as it does, just enough of anatomy and surgery to enable them to do their work with interest and intelligence. Commencing with a sketch of the structure of the skeleton, the author quickly turns aside to discuss the general process of inflammation and of the healing of wounds, a matter without some knowledge of which the surgical details, which arise continually as the book proceeds, could not be understood by a reader who was new to the subject. Then fractures and the various injuries and diseases of joints are considered, and in the same way going through the different parts and organs of the body, the necessary anatomical details are first given, and then, in each case, the accidents and diseases to which they are liable are described. Lastly come some useful chapters on burns, scalds, ulcers, etc., on bandaging, and on instruments.

The book keeps well to its text, and the author has resisted the temptation to stray into popular physiology, and has thus been able to find room for a surprising amount of surgical information, considering the space at his disposal.

It is worth while adding that throughout its pages surgery is treated from an antiseptic and aseptic platform, and that modern methods of treatment are described. The book is well illustrated throughout, and is likely to prove of great use to those for whom it is intended.

Chums: A Magazine for Boys. (Cassell and Co.)

With the number of August 26th began a new volume of this very excellent boy's paper. Therein is contained the first chapter of a story of thrilling interest, entitled "Gerard's Jungle," by G. M. Fenn. Two columns are devoted to the experiences of famous men in the matter of amusement. Fac-similes of the autographs of some of the writers, such as Sir Edwin Arnold, Mr. Hall Caine, Wilson Barrett, and others; true fire brigade yarns, and an interview with the famous cricketer, Prince Ranjitsinhji, and many other interesting items, will render the new volume very popular with young readers. "Chums" is a wonderful outlay for one penny. We hope to see some more numbers.

Cassell's Family Magazine.

"Cassell's Family Magazine" for September contains, besides the current story, some very interesting articles. "The Cycling Academy" is an amusing sketch, which will appeal to a large public. The illustrations are very funny. "Lady Benson's House Party" is a good up-to-date story, and the pages devoted to "Paying Occupations for Gentlewomen" will be found very useful. The beautiful English Home, Taplow Court, near Maidenhead, is charmingly illustrated.

### Notes and Queries.

The contents of the Editor's Letter-box have now reached such unwieldy proportions that it has become necessary to establish a hard and fast rule regarding Answers to Correspondents. In future, all questions requiring replies will continue to be answered in this column without any fee. If an answer is required by letter, a fee of half-a-crown must be enclosed with the note containing the enquiry. We are always pleased to help our numerous correspondents to the fullest extent, and we can trust them to sympathise in the overwhelming amount of writing which makes the new rules a necessity. Every communication must be accompanied by the writer's name and address, otherwise it will receive no attention.

### Queries.

(159) *Pensions for Hospital Secretaries.*—Can you tell me if there is any institution for providing secretaries with pensions or sick pay, after the manner of the Nurses' Pension Fund?—*Secretary.*

(160) *Sanitary Inspectorship.*—What subjects should one study to obtain a certificate as sanitary inspector?—*Male Nurse.*

(161) *Information Wanted.*—Can you tell me (1) Is it true that people are in the habit of raising money by selling their bodies to hospitals for purposes of dissection after death? (2) Do you think it is fair for hospital matrons to refuse women of 28 or 30 years of age as probationers because they are too old? (3) Do you consider the training in London hospitals superior to that in provincial hospitals?—*X X X.*

(162) *Nursing Abroad.*—Please give me the address in London of the Hon. Secretary Colonial Nursing Association.—*Nurse L.*

(163) *St. Moritz.*—Please tell me if there is a nursing institute for private nurses at St. Moritz, or if there would be any opening there for a trained nurse working on her own account?—*Engadine.*

(164) *Nursing Abroad.*—I understand that European nurses are about to be appointed at the Aden Hospital. To whom should I apply for information?—*Miss A. F.*

(165) *Advice Wanted.*—I am anxious to find a suitable place to which to take a lady who is lame and depressed. Can you tell me if we could find clean and really comfortable quarters in some part of the New Forest, where drives would be obtainable, and easy walks. The name of a good hotel or lodgings would be a great help.—*Enquirer.*

(166) *Cottage Hospitals.*—Where can I get a list of English cottage hospitals, and what would be the best way to set about obtaining an appointment as charge nurse or matron in one?—*Trained Nurse.*

(167) *Sterilising Milk.*—What is the best method of sterilising milk?—*Nurse S.*

(168) *Training.*—Will you please advise me in the following circumstances: I am a married woman without children. My husband is in Africa, where I am to join him in two or three years. In the meantime, as I know a knowledge of nursing is very essential for one whose home will be in the interior of Africa, I wish to train as a nurse, and would be glad if you would advise me what to do.—*Anxious.*

### Answers.

(159) *Pensions for Hospital Secretaries (Secretary).*—Write to the Secretary, Royal National Pension Fund for Nurses, 28, Finsbury Pavement, E.C. All hospital officials are eligible for participation in the benefits of this fund.

(160) *Sanitary Inspectorship (Male Nurse).*—See answer to query under this heading in last week's Hospital in this column.

(161) *Information Wanted (X X X).*—We have heard of people to whom such an idea has occurred, but surely you do not imagine that hospital authorities would enter into such negotiations. (2) It is not customary for matrons to refuse as probationers women of 28 and 30, unless there should be some other disqualification than that of age. Thirty-five is the usual limit, but in some hospitals it has been extended lately. (3) The training given to probationers at many provincial hospitals is excellent, and by no means to be considered inferior to London hospitals. In hospitals were there is no medical school duties devolve upon the nurses which would otherwise be done by the students, and for this reason many nurses prefer the experience to be gained in provincial institutions.

(162) *Nursing Abroad (Nurse L.).*—The address of the Colonial Nursing Association is the Imperial Institute, S.W. Read the letter from the hon. secretary in The Hospital Nursing Supplement for August 15th. There are more applications than can be replied to.

(163) *St. Moritz (Engadine).*—We do not know of any institute for private nurses at St. Moritz. If you write to Dr. Tidey, Villa Poletti, St. Moritz Bad, we feel sure he will be kind enough to tell you if, in his opinion, there is any opening for a nurse working on her own account.

(164) *Nursing Abroad (Miss A. F.).*—You had better write to the Medical Superintendent of the European General Hospital, Aden.

(165) *Advice Wanted (Enquirer).*—You will find the hotel at Lyndhurst very comfortable, and there are good lodgings to be had both there and at Brockenhurst, which latter place may possibly be the best for your purpose. Mrs. Dukes, Woodbine Cottage, would recommend you to rooms. There are abundance of charming walks and drives in the neighbourhood.

(166) *Cottage Hospitals (Trained Nurse).*—You will find a full list in Burdett's "Cottage Hospitals" (Scientific Press, 428, Strand, W.C.). You should watch the advertisements in our columns, or advertise yourself. Certainly with your experience you should be able to obtain the post you desire.

(167) *Sterilised Milk (Nurse S.).*—You will find a full description of the process in Dr. Murray Braidwood's "Mother's Help and Guide" (Scientific Press, 428, Strand, W.C.), on page 142. The apparatus there recommended may be obtained from Messrs. Maw, Son, and Thompson, Aldersgate Street, E.C.

(168) *Training (Anxious).*—You are certainly wise to gain some knowledge of nursing before going out to Africa. You do not say if you are prepared to pay for your training or not. As perhaps you know, hospitals will not, as a rule, accept married women as regular or paid probationers, these having to agree to remain in the service of the hospital for a certain number of years. You could enter a London or provincial hospital as a paying probationer for three months (the pay is usually £13 13s. for that period), and you should write to the matrons of various general hospitals for particulars of the training, and state your circumstances and wishes. You will find a list of hospitals in "How to Become a Nurse," Scientific Press, 428, Strand, W.C., from which to select.

## Figures and Tables

**Fig. 35. f1:**
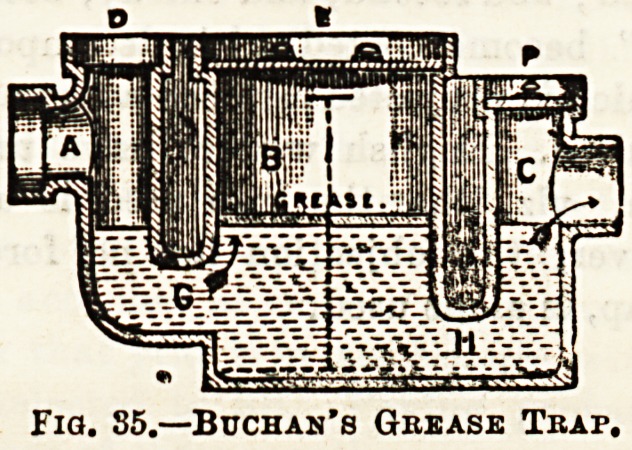


**Fig. 36. f2:**